# DDX39B contributes to the proliferation of colorectal cancer through direct binding to CDK6/CCND1

**DOI:** 10.1038/s41420-022-00827-7

**Published:** 2022-01-19

**Authors:** Haonan Zhang, Chengcheng He, Xuxue Guo, Yuxin Fang, Qiuhua Lai, Xinke Wang, Xingzhu Pan, Haolin Li, Kaiwen Qin, Aimin Li, Side Liu, Qingyuan Li

**Affiliations:** 1 Guangdong Provincial Key Laboratory of Gastroenterology, Department of Gastroenterology, Nanfang Hospital, Southern Medical University, Guangzhou, Guangdong China; 2 Department of Gastroenterology, The Third Affiliated Hospital of Guangzhou Medical University, Guangzhou, Guangdong China; 3 Nanfang Hospital (The First School of Clinical Medicine), Southern Medical University, Guangzhou, Guangdong China

**Keywords:** Oncogenes, Colon cancer

## Abstract

DDX39B (also called UAP56 or BAT1) which is a kind of DEAD-box family helicase plays pivotal roles in mRNA binding, splicing, and export. It has been found upregulated in many kinds of tumors as an oncogene. Nevertheless, the underlying molecular mechanisms of DDX39B in the proliferation of human colorectal cancer (CRC) remain fairly elusive. In our study, function experiments including the CCK8 and colony formation assay revealed that DDX39B facilitates CRC proliferation in vitro. DDX39B knockdown cells were administered for the orthotopic CRC tumor xenograft mouse model, after which tumor growth was monitored and immunohistochemistry (IHC) was performed to prove that DDX39B can also facilitates CRC proliferation in vivo. Flow cytometry demonstrated that DDX39B promotes the proliferation of CRC cells by driving the cell cycle from G0/G1 phase to the S phase. Mechanistically, RNA-binding protein immunoprecipitation-sequencing (RIP-seq) confirmed that DDX39B binds directly to the first exon of the CDK6/CCND1 pre-mRNA and upregulates their expression. Splicing experiments in vitro using a RT-PCR and gel electrophoresis assay confirmed that DDX39B promotes CDK6/CCND1 pre-mRNA splicing. Rescue experiments indicated that CDK6/CCND1 is a downstream effector of DDX39B-mediated CRC cell proliferation. Collectively, our results demonstrated that DDX39B and CDK6/CCND1 direct interactions serve as a CRC proliferation promoter, which can accelerate the G1/S phase transition to enhance CRC proliferation, and can offer novel and emerging treatment strategies targeting this cell proliferation-promoting gene.

## Introduction

Colorectal cancer (CRC) has become the third most common cause of cancer and the leading cause of cancer death worldwide to threaten our lives and health [1]. The dysregulation of cell proliferation and cell cycle is an important biological feature in the development of colorectal cancer [2]. The cell cycle-related genes are important to maintain the normal cell division and their abnormal expression will lead to tumorigenesis [3]. Many critical checkpoints are responsible for replication errors detecting and arresting the cell cycle until repairs are completed between the different phases of the cell cycle [4]. One of the most important and widely studied is the G1/S checkpoint [5]. Several kinds of cyclin-dependent kinases (CDKs) and their cyclin partners can form the complexes to play a fundamental role in the regulation of G1/S checkpoints, such as the cyclinD1 (CCND1)/CDK4/CDK6 or the cyclin E (CCNE)/CDK2 [6, 7].

More and more cancer driver events are thought to be directed by perturbations of RNA expression or processing [8]. DDX39B (also called UAP56), is a member of the DEAD-box (DDX) RNA helicase family which is named after their Asp-Glu-x-Asp/His motifs. They can be involved in almost all the RNA processes such as the mRNA capping, splicing, poly-adenylating to be the mature mRNA and export [9]. On the one hand, DDX39B is well known to promote assembly of the spliceosome [10] and multiple aspects of RNA metabolism [11]. On the other hand, DDX39B can be retained on spliced mRNA in an exon junction complex (EJC), which is deposited ∼20 nucleotides upstream of the exon and it can subtly be coordinated by the transcription and export (TREX) complex (THO–UAP56/DDX39B–ALYREF) at the 5’end to facilitate the export of the spliced mRNA to the cytoplasm [12]. Meanwhile, DDX39B has been found to be elevated in diverse cancers. It has been reported that DDX39B can recruit ALYREF onto BRCA1 mRNA to facilitate DNA repair by homologous recombination to suppress the chemosensitivity in ovarian cancer [13]. Removal of intron sequences from eukaryotic messenger RNA precursors that is called Alternative splicing (AS) is carried out by the spliceosome, a complex assembly of five small nuclear RNAs and a large number of proteins. DDX39B possesses ATP-dependent RNA unwinding/helicase activity that can drive the spliceosome assembly and modulate specific RNA structural rearrangements [14, 15]. AS plays a vital role in forming mature mRNAs from pre-mRNAs, which induces generation for variation of transcript and diversity of proteome. It is highly correlated with tumorigenesis [16–18]. DDX39B can produce abnormal expression of spliceosomes in breast cancer to promote its invasiveness [19] and regulate androgen receptor splice variant generation that is associated with resistance to androgen deprivation therapy in prostate cancer patients through the aberrant AS [20]. Additionally, it can be used for identifying the signatures of AS in kidney renal clear cell carcinoma [21].

In our previous study, we have confirmed that DDX39B can upregulate the expression of FUT3 through the aberrant AS and then promoting the fucosylation of TGFβR-I, which subsequently enhances activation of the TGFβ/SMAD2 signaling pathway to facilitate the invasion and metastasis of CRC [22].

And our study focused on the mechanism of DDX39B in the proliferation of colorectal cancer, we found that DDX39B can bind directly to the exons of CCND1/CDK6 mRNA to upregulate their expression and promote the CRC cell proliferation as an oncogene. These results provide novel insight into the expression of DDX39B within CRC and provide a new therapeutic strategy targeting the cell cycle in CRC.

## Results

### The expression levels of DDX39B in colorectal cancer tissues and its relationship with prognosis

**Fig. 1:**
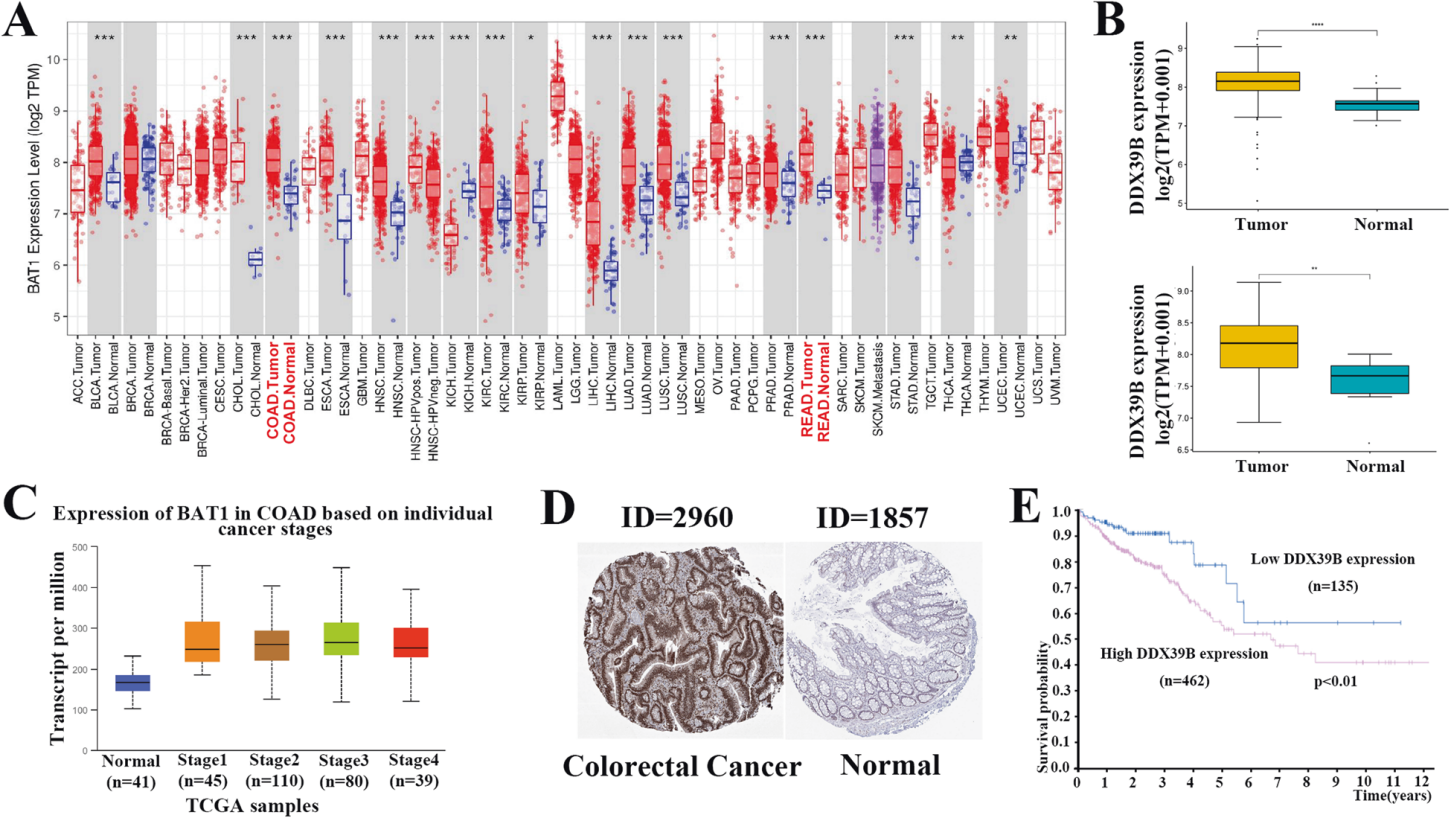
The expression levels of DDX39B in colorectal cancer tissues and its relationship with prognosis. **A** mRNA expression levels of DDX39B were explored in different cancer types via TIMER. **B** The expression of DDX39B is increased in the colon adenocarcinoma (COAD) and rectal adenocarcinoma (READ) compared to the normal tissues. **C** Expression levels of COAD were explored based on individual cancer stages. **D** Immunohistochemistry staining of DDX39B in colorectal cancer and normal tissues were explored in the HPA (Human Protein Atlas) database. **E** Kaplan–Meier curve of OS of COAD were explored based on the expression of DDX39B. Patients were separated either Low (blue, *n* = 135) or High (red, *n* = 462) expression of DDX39B in the HPA database. *P* value was calculated in the plot.

The mRNA expression levels of DDX39B in different cancer types were explored by TIMER, it is worth mentioning that its expression levels were higher in most cancer types than the normal tissues including the COAD and READ (Fig. 1A). And the results that we used the data from the Cancer Genome Atlas (TCGA) database to analyze suggest the same (Fig. 1B). Meanwhile, according to the TCGA datasets, we found the different expression levels of DDX39B in different colorectal cancer stages by UALCAN (Fig. 1C). Subsequently, we wanted to explore the relationship of the DDX39B protein expression levels between the colorectal cancer tissues and normal tissues, so we found some Immunohistochemistry staining images of DDX39B in the HPA (Human Protein Atlas) database, we can obviously find that the colorectal cancer tissues showed the higher staining intensity of DDX39B which is mainly located in the nucleus than the normal tissues (Fig. 1D). Additionally, we found the patients that have higher DDX39B expression levels have a poorer prognosis than the lower by the HPA (Fig. 1E).

### DDX39B affects the proliferation of CRC in vitro and in vivo

**Fig. 2:**
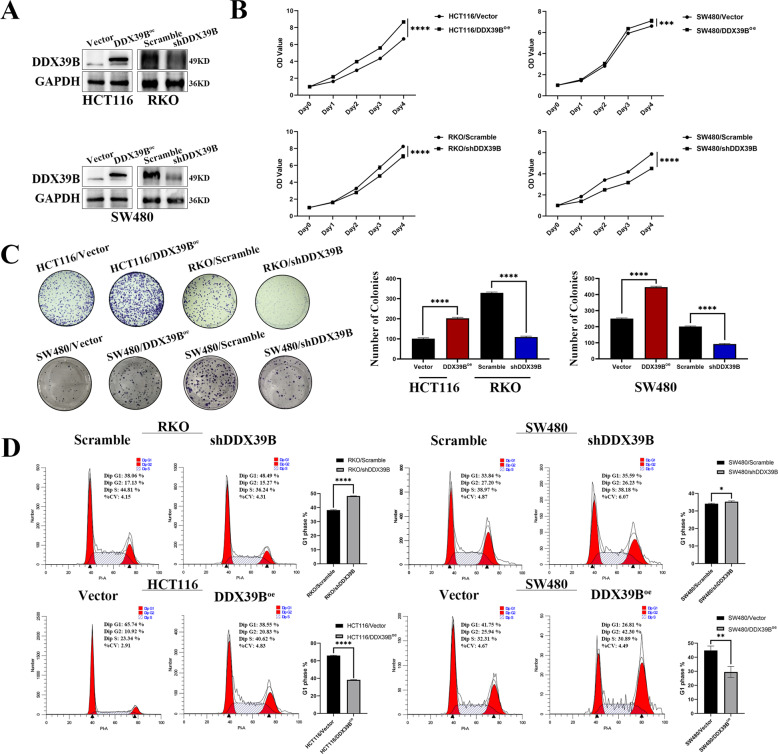
DDX39B promotes the proliferation of colorectal cancer in vitro. **A** Transfected efficiency was determined using western blotting in HCT116, SW480, and RKO cells with overexpressed or silenced DDX39B. **B** CCK8 assay was used for exploring the effect of overexpression of DDX39B on the proliferation of colorectal cancer in HCT116, SW480 cell lines; and the effect of inhibition of DDX39B in SW480, RKO cell lines (one-sample *t*-test). **C** Colony-forming assay was used for detecting the effect of overexpression and inhibition of DDX39B on the proliferation of colorectal cancer in HCT116, SW480 and RKO cell lines, 6-well plate, 1000 cells per well. The number of colonies were calculated in the form of a bar chart, by (one-sample *t*-test) pairwise. **D** Flow cytometry was used to detect the cell cycle of RKO/Scramble, RKO/shDDX39B; SW480/Scramble, SW480/shDDX39B; HCT116/Vector, HCT116/DDX39B^oe^; SW480/Vector, SW480/DDX39B^oe^ groups with PI staining. Bar graph were used to show the G1 phase of cell cycle (one-sample *t*-test). The experiments were performed in three times.

We first examined the transfection efficiency in CRC cells using western blotting in HCT116, SW480, and RKO cells with overexpressed or silenced DDX39B (Fig. 2A). To validate the malignant biological function of DDX39B in CRC cells in vitro, we performed the CCK8 assays and colony-forming assays, then we found that overexpression of DDX39B will markedly increase colony number and optical density (OD) value compared with the control groups. In addition, knockdown of DDX39B will decrease colony number and OD value compared with control groups (Fig. 2B, C). So, we detected the effect of the DDX39B on the cell cycle by the flow cytometry in the RKO/Scramble, RKO/shDDX39B; SW480/Scramble, SW480/shDDX39B; HCT116/Vector, HCT116/DDX39B^oe^; SW480/Vector, SW480/DDX39B^oe^ groups, the results showed the percentage of the G1 phase in the DDX39B overexpression groups is lower than the control groups, and additionally, the DDX39B knockdown groups have the opposite result (Fig. 2D).

**Fig. 3:**
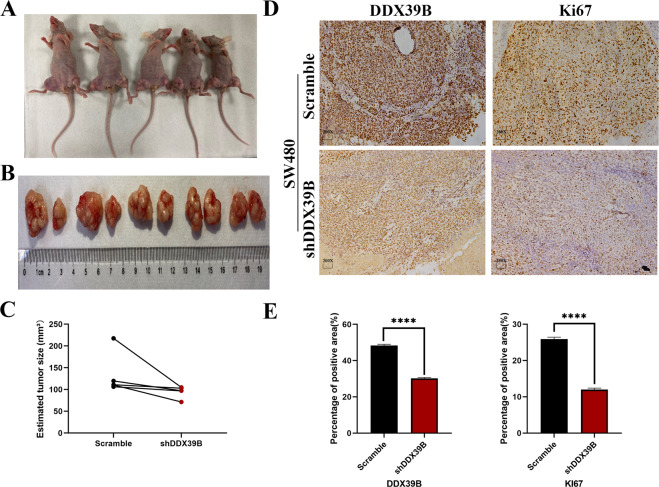
DDX39B promotes the proliferation of colorectal cancer in vivo. **A** SW480/Scramble and SW480/shDDX39B cells were injected into nude mice. **B** Images of the subcutaneous tumors that were injected into the nude mice were obtained. **C** the weight of the tumors were estimated by the Scattergram (paired samples *t*-test). **D**, **E** Immunohistochemistry staining images of DDX39B in tumors were obtained then the positive area was calculated by the ImageJ, and the bar chart was used to demonstrate the DDX39B and Ki67 expression (one-sample *t*-test).

Aiming to detect the effect of DDX39B on proliferation in vivo, we used the nude mice xenograft models that were injected with the DDX39B knockdown cells and the control cells. Based on the results after 20 days, we confirmed the weight of the tumors in the DDX39B knockdown groups is lower than the control groups (Fig. 3A–C). Then the subcutaneous tumor samples from the nude mice were obtained, and we used the immunohistochemistry staining to detect the protein expression levels of Ki67 that is strongly associated with the proliferation [23] and we discovered the DDX39B knockdown groups have the lower Ki67 staining intensity than that in control groups (Fig. 3D, E). So these findings are consistent with the concept DDX39B can affect the proliferation of colorectal cancer in vitro and in vivo.

### DDX39B can elevate the expression of CDK6/CCND1

**Fig. 4:**
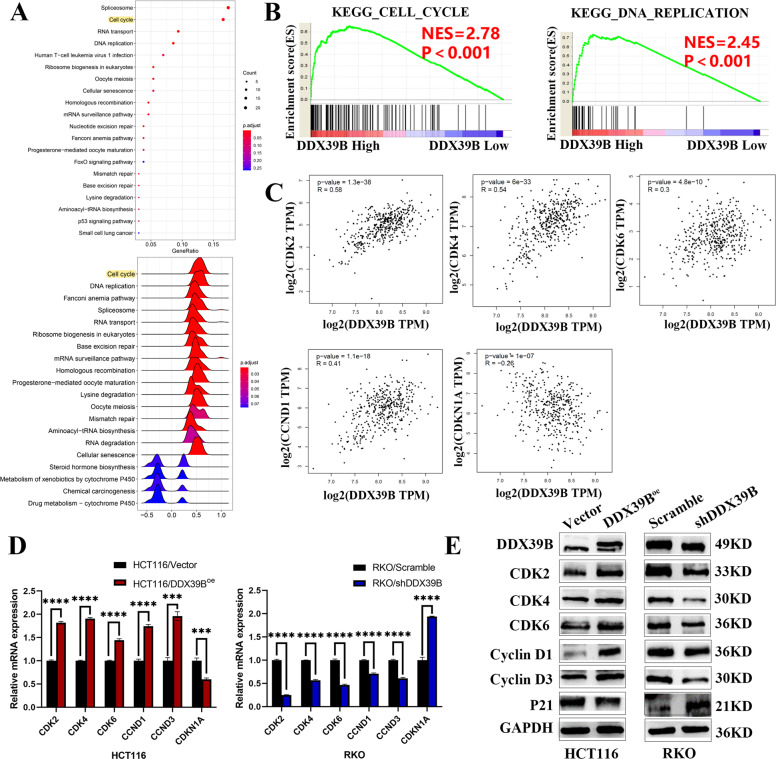
DDX39B can promote the expression of CDK6/CCND1. **A** The cell function enrichment analysis with DDX39B was presented via GO. **B** Enrichment of cell cycle and DNA replication with DDX39B expression in CRC was shown by enrichment analysis of GSEA. **C** The correlation between the DDX39B and the related proteins of cell cycle was explored via the GEPIA. **D** The bar chart was used to show the relative mRNA expression levels of cell cycle-related gene in RKO NCSH/B5, HCT116 NC + /B + by the real-time PCR (one-sample *t*-test). **E** The western blot was used to detect the expression of the cell cycle-related proteins in HCT116/Vector, HCT116/DDX39B^oe^; RKO/Scramble, RKO/shDDX39B.

To demonstrate the mechanism of the DDX39B to the proliferation promotion, the relationship between the DDX39B and cell function was explored by the GO—Cellular components enrichment analysis (Fig. 4A). The results revealed the DDX39B is highly correlated with the cell cycle, and the Gene set enrichment analysis (GSEA) showed similar results (Fig. 4B). Meanwhile, we used the GEPIA website to execute some correlation analysis, and we found the expression of DDX39B is positively correlated with the CDK2, CDK4, CDK6, CCND1; and negatively correlated with the CDK1NA(P21) (Fig. 4C). So the real-time PCR was performed to detect the mRNA expression levels in the RKO Scramble/shDDX39B, HCT116 Vector/DDX39B^oe^ cell lines, the results showed the expression levels of the CDK2, CDK4, CDK6, CCND1 were upregulated with the overexpression of the DDX39B and inhibited with the DDX39B knockdown. The expression of the CDK1NA (P21) which is responsible for the limit of the cell proliferation showed opposite results [24] (Fig. 4D). And the western blot was used to prove the similar results in the protein levels (Fig. 4E).

### DDX39B promotes the cell proliferation of colorectal cancer via targeting the CDK6/CCND1

**Fig. 5:**
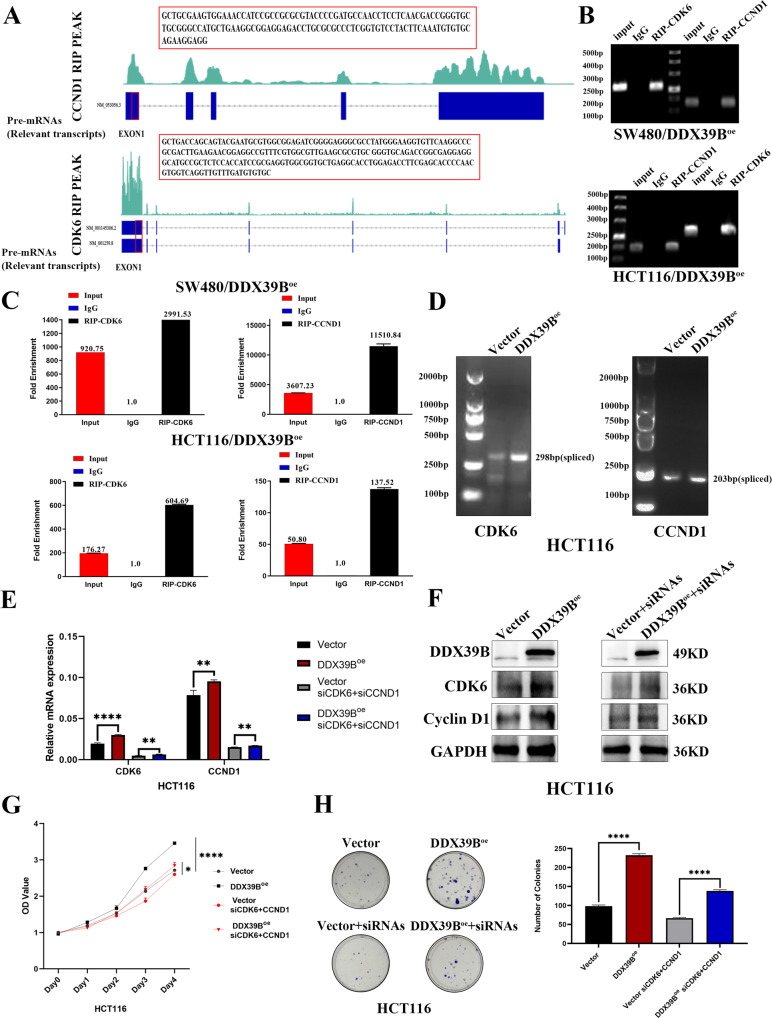
DDX39B promotes the cell proliferation of colorectal cancer via targeting the CDK6/CCND1. **A** RIP-seq of SW480 and HCT116/DDX39B^oe^ showed that DDX39B binds directly to multiple sites of CDK6 and CCND1 pre-mRNA (red box: a partial sequence of the exon1, validated by RIP). **B**, **C** RIP-PCR assay was used to validate the binding of DDX39B to the CDK6/CCND1. **D** RT-PCR and agarose gel electrophoresis assay showed the splicing effect of DDX39B on CDK6/CCND1 pre-mRNA. **E** The relative mRNA expression levels of CDK6 and CCND1 gene in DDX39B ectopic overexpression siCDK6/CCND1 HCT116 cell line and its control by the real-time PCR (one-sample *t-*test). **F** CDK6 and CCND1 protein expression levels of DDX39B ectopic overexpression siCDK6/CCND1 HCT116 cell line and its control. **G** CCK8 assay on DDX39B ectopic overexpression siCDK6/CCND1 HCT116 cell line and its control, by one-sample *t*-test. **H** Colony formation assay on DDX39B ectopic overexpression siCDK6/CCND1 HCT116 cell line and its control, 12-well plate, 400 cells per well. The bar chart demonstrated the analysis of colony numbers by one-sample *t*-test.

**Fig. 6:**
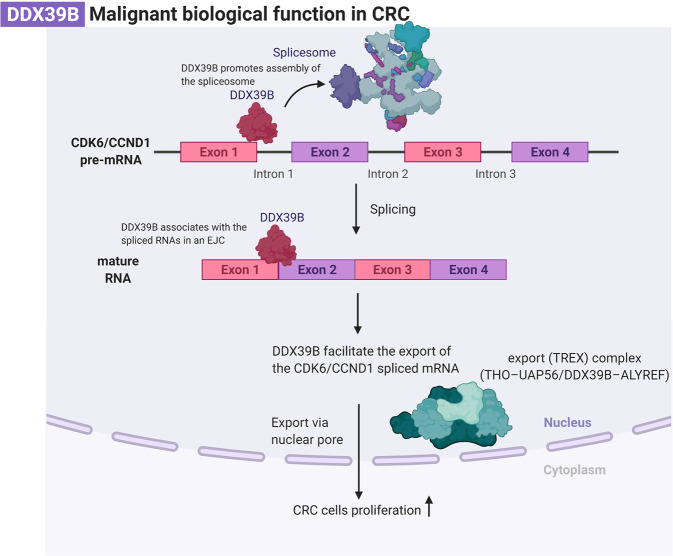
Hypothesized molecular mechanism of DDX39B in proliferation of CRC. This molecular mechanism figure is created with BioRender.com.

To further explore how the DDX39B influences the cell cycle-related protein and the direct binding role of DDX39B to the CDK6/CCND1, RNA-binding protein immunoprecipitation-sequencing (RIP-seq) was performed. As is shown in the picture (Fig. 5A), DDX39B binds directly to multiple possible loci of CDK6/CCND1 pre-mRNA, and the peaks are mainly located in the exons. Then, we used RIP (sequences are labeled in the Fig. 5A red box) to confirm that DDX39B could bind to the first exon of CDK6/CCND1 (Fig. 5B, C). To validate the splicing effect of DDX39B, Semi-quantitative RT-PCR and agarose gel electrophoresis assay were performed to assess the spliced CDK6/CCND1. As shown in Fig. 5D, mature CDK6/CCND1 mRNA (spliced) was significantly increased in HCT116 cells with DDX39B overexpression. To further validate the effect of DDX39B on its downstream directly binding CDK6 and CCND1 genes, we performed the real-time PCR and western blot to detect the mRNA and protein expression levels of CDK6 and CCND1 genes in siCDK6 and siCCND1 HCT116 cells after transfection with DDX39B overexpressing or the Vector lentivirus (Fig. 5E, F). These data indicated the CDK6 and CCND1 expression levels were still upregulated through the DDX39B overexpression. The CCK8 assays and colony-forming assays were also performed to explore whether knockdown of the CDK6 and CCND1 can reverse the phenotype of the DDX39B overexpression cells. And our results showed that the DDX39B ectopic overexpression siCDK6/CCND1 HCT116 cell line have lower OD values and fewer colony-forming numbers than its control (Fig. 5G, H). This phenotype demonstrated that not only overexpression of DDX39B can promote the proliferation of the colorectal cancer cells but also that can be reversed after knockdown of the CDK6 and CCND1. In summary, this important finding further emphasized DDX39B can directly bind to its downstream CDK6 and CCND1 genes and upregulate their expression levels to promote the proliferation of the colorectal cancer cells (Fig. 6).

## Discussions

The DDX family (DEAD-box (Asp-Glu-Ala-Asp) family), a large family of ATP-dependent RNA helicases, has an abundant function in RNA metabolism that can be implicated in some aspects of crucial cellular processes, such as apoptosis, innate immune response, viral replication, cell cycle progression and tumorigenesis [25]. DDX39B, one of the DEAD-box family of RNA-dependent ATPases that mediate ATP hydrolysis during pre-mRNA splicing. It plays an important role in mRNA export from the nucleus to the cytoplasm and It is a fundamental splicing factor that is necessary for the association of U2 small nuclear ribonucleoprotein with pre-mRNA [26]. Consequently, abnormal expression of the DDX39B will cause many adverse effects on mRNA processing and export. The function of the related proteins will also be influenced. In recent years, the role of DDX39B in cancer has been more and more studied, but the mechanism of DDX39B in promoting the proliferation of CRC has not been elucidated. In our early work, we found overexpression of DDX39B will promote CRC proliferation. This phenomenon suggested that the aberrant regulation of DDX39B may contribute to the development of RC as an oncogene. It is worth exploring the underlying key mechanism behind.

Most protein-coding mRNAs in Eukaryotic cells need to undergo a series of mRNA processing including capping at the 5’end, splicing to remove introns, and polyadenylation at the 3’end. The interaction of the mRNA with these cellular machines is determined and regulated by a diverse set of RNA-binding proteins, such as the TREX complexes. They are taken together to make up the mRNA–protein complexes (mRNPs). When the complex is assembled, then they will be exported from the nucleus to the cytoplasm to be translated and perform their function [27]. Eukaryotic mRNA transport is associated with gene expression steps, such as mRNA precursor splicing. As a core export factor, DDX39B has been reported that it is essential for spliceosome assembly [28] and can readily associate with the spliced RNAs in an EJC at each exon-exon junction or form TREX complexes containing multisubunit THO complex, the DEXD-box RNA helicase DDX39B, and an RNA export adapter, such as ALYREF on the first exon to help the mRNA export [29–31]. Therefore, we supposed overexpression of DDX39B might be retained on the first exon of some downstream molecules through forming relevant functional complexes to lead to the malignant biological behavior of the normal cells and promote the aberrant proliferation of CRC. In our study, we use the RIP-seq to find the specific binding site of DDX39B on the pre-mRNA of CCND1/CDK6, and the results showed that the DDX39B-binding motif is enriched with AG/GC which is a part of intron splice junction [32].

The defining feature of the living cells is reproduction, a complex regulatory program which is called the cell cycle. The cell proliferation cycle refers to the time interval from the end of the last cell division to the end of this division, it can be divided into four consecutive phases according to the characteristics of different phases: G1 phase (Gap 1), S phase (Synthesis phase), G2 phase (Gap 2) and M phase (Mitotic phase) [33, 34], When the cell receives the mitogenic signal, the cell will exit the G0 phase and enter the G1 phase to start the cell cycle. And orderly changes in Cyclin/CDK complex are the driving force of the cell cycle. It is through various cyclins and cyclin-dependent protein kinases (CDK) that cells could orderly catalyze the phosphorylation and dephosphorylation of a series of target proteins, and then regulate the activity of the cyclin/CDK complex to achieve the control of cell division [35].

The CCND1 and CDK4/6 complex will shuttle between the nucleus and cytoplasm when it receives the mitogenic signal that can activate the RAS signaling cascades, then the cyclin D1 will be activated to assemble with the CDK4/6 and transported into the nucleus to phosphorylate the Rb and inactivate it, so the E2F will dissociate from it to promote its downstream gene transcription that is necessary for the S phase DNA replication [36] such as the c-myc [37] and increase the cyclin E that can bind to the CDK2 to impel the cell cycle into the S phase [38]. Failure of any part of the cell cycle could cause the cell cycle to get out of control and gradually develop into cancer. The key finding in our study is that DDX39B can directly bind to the first exon of CCND1/CDK6 and regulate cell proliferation by the effect on the cell cycle.

Our study was mainly focused on the key mechanism of DDX39B in the proliferation of CRC. But interestingly, some studies have documented that export activity of DDX39B is dosage-sensitive and both the knockdown and overexpression of DDX39B will inhibit the export of the mRNA, this contradictory manifestation raises a question how do overexpression of DDX39B enhance translation, even if it inhibits mRNA export [39]. One possible explanation is DDX39B can elevate the levels of preribosomal RNA by regulating its stability and transcription [40], and thus our study has some limitations. We just proved the DDX39B can bind directly to the first exon of the CCND1/CDK6 and upregulate their expression by assisting their splicing and export, but the concrete mechanism is still unclear.

On the basis of our data and the characteristics of the unlimited proliferation of tumors. To develop some drugs targeting G1/S transition, such as CCND1/CDK4/6 complex, is a significant way to prevent the progression of the tumor. At present, some cell cycle blockers have been approved for clinical use, such as the CDK4/6 inhibitors: Palbociclib, abemaciclib, and ribociclib that are different from the first generation of the CDK4/6 pan-inhibitors which have toxicity to the normal cells and have been used for multiple cancers treatment [41, 42]. As the CCND1/CDK6 are the downstream effector of the DDX39B, the drug targeting DDX39B may become an effective therapeutic strategy in CRC. In conclusion, DDX39B acts as a colorectal cancer promoter through its effect on the CDK6/CCND1, which can promote the G1/S phase transition to enhance CRC proliferation. Our study provided a new insight into the role of DDX39B in tumorigenesis and more and more new drugs aimed to block the cell cycle progress will be needed.

## Methods

### Bioinformatics analysis

The data of different expression levels of BAT1 in different cancer types were obtained from the database Tumor Immune Estimation Resource (TIMER). DDX39B expression was analyzed with R studio (R version 4.0.3) in paired COAD/READ and normal tissues (samples are from TCGA Database). The database UALCAN was used to explore the expression levels of BAT1 in COAD based on individual cancer stages. The survival analysis curve between the high and low expression of DDX39B groups and immunohistochemistry images of DDX39B protein expression level were obtained from the Human Protein Atlas (HPA). The GO and GSEA Enrichment analysis was used to explore the most likely cell biological function of DDX39B. The GEPIA website was used to explore the correlation between the DDX39B and the cell cycle-related protein.

### Cell culture

Human CRC cell lines (including SW480, HCT116, RKO) were purchased from the Cell Bank of Type Culture Collection (CBTCC, China Academy of Sciences, Shanghai, China). And all cells were maintained with Dulbecco’s modified Eagle’s medium (DMEM) (Gibco) supplemented with 10% certified fetal bovine serum (Biological Industries) in the thermal incubator at 37 °C and 5% CO2.

### Plasmid construction, small interfering RNA, and lentiviral construction

Small interfering RNA and plasmid transfection were carried out by jetPRIME (Polyplus-transfection, France). The siRNAs of DDX39B, CDK6, CCND1, and control group were purchased from the GenePharma (Shanghai, China). They were transfected into SW480 and RKO cells according to the manufacturer’s instructions. The lentivirus human DDX39B construct was generated by cloning PCR-amplified full-length DDX39B cDNA (NM_004640). The DDX39B shRNA sequence was selected from siDDX39B_2. The DDX39B, shRNA, and control lentiviruses (Lianfeng Technology, Shanghai, China) were transfected into RKO, SW480, and HCT116 cells following the manufacturers’ instructions. Then, SW480 and RKO cells with DDX39B knockdown were cultured in a medium with blasticidin (10 μg/ml) (Yesen, China) while SW480 and HCT116 cells with overexpressed DDX39B were cultured in medium with puromycin (2 μg/ml) (BioFroxx, Germany).

### CCK8 and colony formation assay

The cells were seeded in 96-wells plates in 1000 cells per well. And after 24 h, the CCK8 reagents (Dojindo, Japan) were added at the concentration of 10% (90ul medium with 10ul CCK8), and after incubation for 1 h, the absorbance of each well was measured at 450 nm with the microplate reader and This experiment will be measured continuously for five days. The target HCT116, SW480, RKO cell lines, and their control groups were seeded into the 6 plates with 500 cells each well. After culturing for 14 days, all the cells were washed twice by the PBS, and fixed by the formaldehyde for 30 min, and stained with hematoxylin for 20 min, finally, the colonies were counted only those with more than 50 cells. All the experiments were repeated three times.

### Animal study

All animal studies were performed with approval from the Institutional Animal Care and Use Committee of NanFang Hospital. The 4 to 5-week-old BALB/c female nude mice were purchased from the Laboratory Animal Services Centre of Guangdong Province for the xenograft tumor model assay. The experimental mice were randomly selected from the healthy mice by the staff of Laboratory Animal Services Centre of Guangdong Province, excluding the mice with different gender, body shape, and health status. The 5 × 10^6^ cells were prepared to be injected subcutaneously into the left flanks (shDDX39B) or right (scramble) of mice. After 20 days, the mice were sacrificed with phenobarbital sodium and the tumors were weighed and embedded with paraffin for immunohistochemistry.

### Total RNA isolation and qRT-PCR

The total RNA was isolated with the RNAiso (Takara) and following the manufacturer’s protocol. And the RNA was used to synthesis cDNA with the mix (AG). Then the SYBR Green PCR Kit (AG) was used to analyze the cDNA samples by the real-time PCR. The qPCR results were analyzed using the Ct values of the amplified samples, and all the data were analyzed by the 2-ΔΔCt that were normalized by the glyceraldehyde-3-phosphate dehydrogenase (GAPDH). The primers were purchased from the Sangon Biotech.

### Western blot and immunohistochemistry (IHC) analysis

The proteins were extracted using radioimmunoprecipitation assay (RIPA) lysis buffer with the 1% PMSF, and the protein concentration was measured using the BCA Protein Assay Kit (beyotime), and the protein samples were added into the sodium dodecyl sulfate-polyacrylamide gel electrophoresis (SDS-PAGE) for separating proteins of the different molecular weights, and then transferred onto polyvinylidene difluoride (PVDF) membranes. Finally, the following antibodies were used to detect the expression of the protein samples: DDX39B (14798-1-AP, proteintech), CDK2 (78B2, Cell Siganling Technology) CDK4 (D9G3E, Cell Siganling Technology), CDK6 (D4S8S, Cell Siganling Technology), Cyclin D1 (2922, Cell Siganling Technology), Cyclin D3 (DCS22, Cell Siganling Technology), P21 (2947, Cell Siganling Technology), GAPDH (60004-1-Ig, proteintech). The immunohistochemistry assay was performed following the following the manufacturer’s protocols (PV-6001, ZSGB-BIO, Beijing, China) and the following antibodies were used: DDX39B (14798-1-AP, proteintech), Ki67 (27309-1-AP, proteintech). And the results were analyzed by the ImageJ to get the percentage of positive areas.

### RNA-binding protein immunoprecipitation (RIP) and RNA-binding

protein immunoprecipitation-sequencing A Magna RIP Kit (Billerica MA, USA, No.17-701) was used for RIP. In all, 2×107SW480/DDX39B cells (per immunoprecipitation) were collected first, according to the detailed specifications of the Magna RIP Kit. Ten microliters of antibody against the DYKDDDDK Tag (#66008–3, Proteintech) were used for RIP. We used a Magna RIP Kit (17-700) to carry out RIP experiments on SW480/DDX39B cells and sent RIP and Input products to RIBO, China. RIP and input products passed quality-control tests and were sequenced on an Illumina platform. The RIP-seq report was presented by Guangzhou RIBO Biotechnology Co., Ltd.

### Flow cytometry

The cells were washed once with PBS (centrifugation at 2000 rpm, 5 min) and collected to adjust the cell concentration to 1×10^6/ml, then taken 1 ml from the cell suspensions. The supernatant was removed after centrifugation. The cells are fixed with 70% ethanol (4 °C, overnight). The ethanol will be washed away by the PBS, and then add the PI staining (BD) following the manufacturer’s protocols, the samples will be analyzed on the flow cytometer. The data were analyzed by the ModFit LT 5.0.

### RT-PCR and agarose gel electrophoresis assay

The RT-PCR was performed following the manufacturer’s protocols (TaKaRa Premix Taq ™ Version 2.0). The sequences of all the primers were listed as follows (5′–3′): CDK6-F: TTACCTGCTCCGCGAGGC, CDK6-R: GCTGCAGAAGCTGGATGGAG, CCND1-F: CGATGCCAACCTCCTCAACG CCND1-R: CCAGGTAGTTCATGGCCAGC. And the DNA samples were added into the 2% agarose gel for separating DNA bands of the different molecular weights, and then the results of DNA electrophoresis were observed on the UV transmission detector.

### statistical analysis

All group variances were compared with similar statistical methods. All data are representative of at least three independent repeats and presented as the mean ± SE. Statistical analysis were analyzed by SPSS 26 and a two-sided test was applied, the raw data applying t-test was normally distributed. Statistical significance was described as follows: *p < 0.05, **p < 0.01, ***p < 0.001.

## Supplementary information

ORCID of Side Liu

## Data Availability

The data that support the findings of this study are available from the corresponding author upon reasonable request.

## References

[1] Siegel RL, Miller KD, Goding Sauer A, Fedewa SA, Butterly LF, Anderson JC (2020). Colorectal cancer statistics, 2020. CA Cancer J. Clin..

[2] Kappel S, Stoklosa P, Hauert B, Ross-Kaschitza D, Borgstrom A, Baur R (2019). TRPM4 is highly expressed in human colorectal tumor buds and contributes to proliferation, cell cycle, and invasion of colorectal cancer cells. Mol. Oncol..

[3] Guo H, Deng H, Liu H, Jian Z, Cui H, Fang J (2021). Nickel carcinogenesis mechanism: cell cycle dysregulation. Environ. Sci. Pollut. Res Int.

[4] Wenzel ES, Singh ATK (2018). Cell-cycle Checkpoints and Aneuploidy on the Path to Cancer. Vivo.

[5] Massagué J (2004). G1 cell-cycle control and cancer. Nature.

[6] Henri P, Prevel C, Pellerano M, Lacotte J, Stoebner PE, Morris MC (2019). Psoriatic epidermis is associated with upregulation of CDK2 and inhibition of CDK4 activity. Br. J. Dermatol..

[7] Yu L, Ye F, Li YY, Zhan YZ, Liu Y, Yan HM (2020). Histone methyltransferase SETDB1 promotes colorectal cancer proliferation through the STAT1-CCND1/CDK6 axis. Carcinogenesis.

[8] Ganini C, Amelio I, Bertolo R, Bove P, Buonomo OC, Candi E, et al. Global mapping of cancers: The Cancer Genome Atlas and beyond. Mol Oncol. 2021;15:2823–40. 10.1002/1878-0261.13056 PMC8564642 34245122

[9] Shen L, Pelletier J. General and Target-Specific DExD/H RNA Helicases in Eukaryotic Translation Initiation. Int J Mol Sci. 2020;21:4402. 10.3390/ijms21124402 PMC7352612 32575790

[10] Shen H, Zheng X, Shen J, Zhang L, Zhao R, Green MR (2008). Distinct activities of the DExD/H-box splicing factor hUAP56 facilitate stepwise assembly of the spliceosome. Genes Dev.

[11] Szymura SJ, Bernal GM, Wu L, Zhang Z, Crawley CD, Voce DJ (2020). DDX39B interacts with the pattern recognition receptor pathway to inhibit NF-kappaB and sensitize to alkylating chemotherapy. BMC Biol..

[12] Kota KP, Wagner SR, Huerta E, Underwood JM, Nickerson JA (2008). Binding of ATP to UAP56 is necessary for mRNA export. J. Cell Sci..

[13] Xu Z, Li X, Li H, Nie C, Liu W, Li S (2020). Suppression of DDX39B sensitizes ovarian cancer cells to DNA-damaging chemotherapeutic agents via destabilizing BRCA1 mRNA. Oncogene.

[14] Eymin B (2021). Targeting the spliceosome machinery: A new therapeutic axis in cancer?. Biochem Pharm.

[15] Sahni A, Wang N, Alexis JD (2010). UAP56 is an important regulator of protein synthesis and growth in cardiomyocytes. Biochem Biophys. Res. Commun..

[16] Cherry S, Lynch KW (2020). Alternative splicing and cancer: insights, opportunities, and challenges from an expanding view of the transcriptome. Genes Dev..

[17] Bonnal SC, Lopez-Oreja I, Valcarcel J (2020). Roles and mechanisms of alternative splicing in cancer - implications for care. Nat. Rev. Clin. Oncol..

[18] Farina AR, Cappabianca L, Sebastiano M, Zelli V, Guadagni S, Mackay AR (2020). Hypoxia-induced alternative splicing: the 11th Hallmark of Cancer. J. Exp. Clin. Cancer Res.

[19] Wang L, Wang Y, Su B, Yu P, He J, Meng L (2020). Transcriptome-wide analysis and modelling of prognostic alternative splicing signatures in invasive breast cancer: a prospective clinical study. Sci. Rep..

[20] Nakata D, Nakao S, Nakayama K, Araki S, Nakayama Y, Aparicio S (2017). The RNA helicase DDX39B and its paralog DDX39A regulate androgen receptor splice variant AR-V7 generation. Biochem Biophys. Res Commun..

[21] Meng T, Huang R, Zeng Z, Huang Z, Yin H, Jiao C (2019). Identification of Prognostic and Metastatic Alternative Splicing Signatures in Kidney Renal Clear Cell Carcinoma. Front Bioeng. Biotechnol..

[22] He C, Li A, Lai Q, Ding J, Yan Q, Liu S, et al. The DDX39B/FUT3/TGFβR-I axis promotes tumor metastasis and EMT in colorectal cancer. Cell Death Dis. 2021;12:74. 10.1038/s41419-020-03360-6 PMC7803960 33436563

[23] Menon SS, Guruvayoorappan C, Sakthivel KM, Rasmi RR (2019). Ki-67 protein as a tumour proliferation marker. Clin. Chim. Acta.

[24] de Azevedo SSD, Ribeiro-Alves M, Cortes FH, Delatorre E, Spangenberg L, Naya H (2020). Increased expression of CDKN1A/p21 in HIV-1 controllers is correlated with upregulation of ZC3H12A/MCPIP1. Retrovirology.

[25] Kukhanova MK, Karpenko IL, Ivanov AV. DEAD-box RNA Helicase DDX3: Functional Properties and Development of DDX3 Inhibitors as Antiviral and Anticancer Drugs. Molecules. 2020;25:1015. 10.3390/molecules25041015 PMC7070539 32102413

[26] Fleckner J, Zhang M, Valcárcel J, Green MR (1997). U2AF65 recruits a novel human DEAD box protein required for the U2 snRNP-branchpoint interaction. Genes Dev.

[27] Khong A, Parker R (2020). The landscape of eukaryotic mRNPs. RNA.

[28] Reichert VL, Le Hir H, Jurica MS, Moore MJ (2002). 5’ exon interactions within the human spliceosome establish a framework for exon junction complex structure and assembly. Genes Dev..

[29] Cheng H, Dufu K, Lee CS, Hsu JL, Dias A, Reed R (2006). Human mRNA export machinery recruited to the 5’ end of mRNA. Cell.

[30] Gromadzka AM, Steckelberg AL, Singh KK, Hofmann K, Gehring NH (2016). A short conserved motif in ALYREF directs cap- and EJC-dependent assembly of export complexes on spliced mRNAs. Nucleic Acids Res.

[31] Puhringer T, Hohmann U, Fin L, Pacheco-Fiallos B, Schellhaas U, Brennecke J, et al. Structure of the human core transcription-export complex reveals a hub for multivalent interactions. Elife. 2020;9:e61503. 10.7554/eLife.61503 PMC7744094 33191911

[32] Abou Alezz M, Celli L, Belotti G, Lisa A, Bione S (2020). GC-AG Introns Features in Long Non-coding and Protein-Coding Genes Suggest Their Role in Gene Expression Regulation. Front Genet.

[33] Kastan MB, Bartek J (2004). Cell-cycle checkpoints and cancer. Nature.

[34] Stamatakos M, Palla V, Karaiskos I, Xiromeritis K, Alexiou I, Pateras I (2010). Cell cyclins: triggering elements of cancer or not?. World J. Surg. Oncol..

[35] Knudsen ES, Pruitt SC, Hershberger PA, Witkiewicz AK, Goodrich DW (2019). Cell Cycle and Beyond: Exploiting New RB1 Controlled Mechanisms for Cancer Therapy. Trends Cancer.

[36] Tchakarska G, Sola B (2020). The double dealing of cyclin D1. Cell Cycle.

[37] Luo Q, Wu X, Chang W, Zhao P, Nan Y, Zhu X (2020). ARID1A prevents squamous cell carcinoma initiation and chemoresistance by antagonizing pRb/E2F1/c-Myc-mediated cancer stemness. Cell Death Differ..

[38] Kar S (2016). Unraveling Cell-Cycle Dynamics in Cancer. Cell Syst..

[39] Luo ML, Zhou Z, Magni K, Christoforides C, Rappsilber J, Mann M (2001). Pre-mRNA splicing and mRNA export linked by direct interactions between UAP56 and Aly. Nature.

[40] Awasthi S, Chakrapani B, Mahesh A, Chavali PL, Chavali S, Dhayalan A (2018). DDX39B promotes translation through regulation of pre-ribosomal RNA levels. RNA Biol.

[41] Goel S, DeCristo MJ, McAllister SS, Zhao JJ (2018). CDK4/6 Inhibition in Cancer: Beyond Cell Cycle Arrest. Trends Cell Biol..

[42] Liu M, Liu H, Chen J (2018). Mechanisms of the CDK4/6 inhibitor palbociclib (PD 0332991) and its future application in cancer treatment (Review). Oncol. Rep..

