# Supplementary material for: DDX39B contributes to the proliferation of colorectal cancer through direct binding to CDK6/CCND1
Source: Cell Death Discov. 2022 Jan 19;8:30. doi: 10.1038/s41420-022-00827-7 (PMC8770491; doi:10.1038/s41420-022-00827-7)
Supplement: Supplementary file 1 — ORCID of Side Liu [file 41420_2022_827_MOESM1_ESM.docx]

Dear Editor,

I’m the corresponding author of the manuscript named DDX39B contributes to the proliferation of colorectal cancer through direct binding to CDK6/CCND1(Manuscript Number: CDDISCOVERY-21-2533R1). First of all, I would like to express my deep apologies for our negligence. Following your suggestions, I tried to link the ORCID of the Professor Side Liu, but I’m not sure whether I linked it correctly, so I want to give you the ORCID of the Professor Side Liu, it’s 0000-0001-8192-988X, I wish it can help.

Yours sincerely,

Dr Qingyuan Li
